# Biomarkers of Neonatal Sepsis: From Being Mere Numbers to Becoming Guiding Diagnostics

**DOI:** 10.7759/cureus.23215

**Published:** 2022-03-16

**Authors:** Sai Sravya Gude, Nikhil Chowdary Peddi, Sravya Vuppalapati, Shravya Venu Gopal, Harshita Marasandra Ramesh, Sai Sreeya Gude

**Affiliations:** 1 Pediatrics, Guntur Medical College, Guntur, IND; 2 Pediatrics, PES Institute of Medical Sciences and Research, Kuppam, IND; 3 Internal Medicine, Kasturba Medical College Mangalore, Mangalore, IND; 4 Internal Medicine and Pediatrics, Kasturba Medical College Mangalore, Mangalore, IND; 5 Internal Medicine, Guntur Medical College, Guntur, IND

**Keywords:** c-reactive proteins(crp), infection, metabolomics, serum biomarkers, neonatal sepsis

## Abstract

Neonatal sepsis is a common cause of neonatal morbidity and mortality. The diagnosis of newborn sepsis is still difficult. Different early objective diagnostic tests or specific signs and symptoms, particularly in preterm infants, make it difficult to diagnose neonatal sepsis. This review article describes biomarkers and their role in the early diagnosis, treatment, and prognosis of neonatal sepsis. It also explores the possible advances and future prospects of these biomarkers. An ideal sepsis biomarker will not only help in the guidance of the use of antibiotics when not needed but also the duration of the course of antibiotics if sepsis is proven. It should also have high sensitivity, specificity, positive predictive value, and negative predictive value. These biomarkers hold a promising position in the management of neonatal sepsis and translate into use in clinical settings. Metabolomics, a diagnostic method based on detecting metabolites found in biological fluids, may open new possibilities in the management of critically ill newborns.

## Introduction and background

Neonatal sepsis is a clinical syndrome of systemic illness accompanied by the bacterium in the first 28 days of life. Neonatal sepsis can be divided into two types: early-onset sepsis, which manifests as respiratory distress within 72 hours of birth, and late-onset sepsis, which manifests as septicemia after 72 hours of birth [1-3].

The reported incidence of neonatal sepsis ranges from one to five cases per 1000 live births, with one to two cases per 1000 live births in term neonates, including both early-onset sepsis (EOS) and late-onset sepsis (LOS). The overall mortality rate is up to 24.4%, but it can be as high as 54% in infants born between 22 and 24 weeks of pregnancy and 30% in those born between 25 and 28 weeks [4].

Various risk factors for a neonate to develop EOS include maternal risk factors like chorioamnionitis, premature rupture of membranes, preterm pregnancy, prolonged rupture of membranes, intrapartum maternal fever, and neonatal risk factors like preterm newborn, low birth weight, fetal distress, low APGAR score, multiple pregnancies [1]. Reduced feed acceptance, respiratory distress, pneumonia, apnea, delayed capillary refill time, cold peripheries, mottling, off colour, feed intolerance, necrotizing enterocolitis, temperature instability (hypothermia and hyperthermia), hypotonia, seizures, bulging fontanels, disseminated intravascular coagulation (DIC), bleeding manifestation, and prolonged jaundice are some of the clinical manifestations of neonatal sepsis [4,5].

In addition, neonatal sepsis leads to severe complications for the newborn like post-infectious encephalopathy, seizures, ventriculomegaly, hydrocephalus, encephalomalacia, brain infarction, neurodevelopmental delay, and sensorial deficits [1].

Sepsis workups are performed on infants who show clinical symptoms of infection, and broad-spectrum antibiotics are started until a clear diagnosis can be made. The gold standard for establishing a definitive diagnosis of newborn sepsis is the isolation of microorganism(s) from blood cultures [6,7].

Many sepsis biomarkers have been studied to determine their utility in the diagnosis of neonatal sepsis. An ideal biomarker should have high sensitivity, high specificity, positive predictive value, and negative predictive value (NPV) [4]. The biomarkers are divided into acute-phase proteins, cell surface antigens, cytokines and chemokines, and soluble adhesion molecules. C-reactive protein (CRP), procalcitonin, serum amyloid A, and hepcidin are acute-phase proteins that are used to diagnose neonatal sepsis. CD64 and CD11b are cell surface antigens involved in the detection of newborn sepsis. Interleukin (IL) IL-1, IL-6, IL-8, tumour necrosis factor (TNF), and soluble TNF receptor (sTNFR) are cytokines and chemokines implicated in the diagnosis of neonatal sepsis. Soluble adhesion indicators concerned with the diagnosis of newborn sepsis are E-selectin and intercellular adhesion markers (ICAM) [5,8-10,11].

This review article briefly describes each of the biomarkers and their role in the early diagnosis, treatment, and prognosis of neonatal sepsis. It also explores the possible advances and prospects of these biomarkers.

## Review

Biomarkers

Complete Blood Count (CBC)

The complete blood count (CBC), differential count, and immature-to-total leukocyte ratio (I:T) have all been widely studied for the diagnosis of infant sepsis. Despite the fact that the CBC has a low predictive value, a series of normal readings can increase the chances of diagnosing bacterial sepsis [4]. CBCs are used in a variety of ways, including obtaining a single CBC or serially, evaluating the total white blood cell count, neutrophil count, immature-to-total ratio, temporal trends, and analyzing red blood cell and platelet size and morphology, in addition to leukocyte morphology [12].

Low white blood count (WBC) and absolute neutrophil counts, as well as a high I:T, are connected to an increased risk of infection. The WBC components of the absolute neutrophil count (ANC) and I:T have also been shown to be more useful for eliminating non-infected newborns than identifying infected infants. Sepsis is diagnosed when the I:T ratio is greater than 0.2. Noninfectious events including labour, prolonged oxytocin induction, and even prolonged crying can all affect the I:T ratio. A total leukocyte count of 5000 to 7500/mm3 can be used to diagnose infant sepsis. When interpreting the leucocyte count, the neonatal gestational age must be taken into account, as the lower limit of ANC reduces as gestational age decreases [13]. For ruling out infant sepsis, the ANC and I:T ratio demonstrated an excellent NPV [4]. 

Neonatal sepsis is more likely when the I:T ratio is greater than 0.2. Perinatal hypoxia, maternal hypertension, labour stress, and prolonged oxytocin induction are all factors that might cause erroneous I:T ratio changes. Neutropenia has been found to be a stronger predictor of infant sepsis than neutrophilia. In conclusion, there are significant limitations to the use of WBC, ANC, and I/T ratio in the diagnosis of infant sepsis. Many infected newborns may have higher numbers of WBC, ANC, and I/T ratio [13].

The lowest limit of normal for ANC at birth in babies >36 weeks gestation is 3500/mm3. The lowest limit of normal for children born between 28 and 36 weeks is 1000/mm3, but it is 500/mm3 for infants born before 28 weeks. Total neutrophil levels rise after delivery and peak around six to eight hours of life. The lower limits of normal for infants born at >36 weeks, 28-36 weeks, and 28 weeks are 7500/mm3, 3500/mm3, and 1500/mm3, respectively. Because total leukocyte counts collected six to 12 hours after birth are more likely to be correct, they are more effective. Factors such as maternal hypertension or neonatal hypoxia might produce neutropenia or an elevated I/T ratio. Also, in the early stages of newborn sepsis, leukocyte counts may be normal. In conclusion, the WBC, ANC, and I/T ratios have major limits in identifying newborn sepsis due to these variances [13].

C-reactive protein

CRP is the most widely used biomarker in laboratory settings for identifying neonatal sepsis. IL-6, IL-1, and tumour necrosis factor are all important stimulatory cytokines. Unfortunately, because CRP has a half-life of 24 to 48 hours and levels require 10 to 12 hours to rise, it is unreliable for early detection of newborn sepsis [4]. 

It is generated in hepatocytes and is transcriptionally regulated by interleukin (IL)-6 and IL-1-beta [14]. CRP is a protein that is produced by the liver and belongs to the category of acute-phase proteins [15]. CRP values require 10-12 hours to change after the onset of infection, has a half-life of 24-48 hours, and is measured on a regular basis to decide the length of antibiotic treatment. CRP is a specific, although late, biomarker for infant infection detection and normal levels indicate that sepsis is not present, and antibiotic treatment should be discontinued [13]. 

CRP levels can be raised by non-infectious illnesses like traumatic or ischemic tissue injury, meconium aspiration syndrome, and hemolysis. CRP levels in preterm neonates normally rise only slightly when they are infected. CRP has a number of disadvantages as a biomarker, including CRP rise in non-infectious situations, the lack of appropriate age-specific reference values, and the impact of birth weight and gestational age on CRP kinetics [13].

Meconium aspiration syndrome (MAS), delayed transition after birth, hemolysis, tissue injury, surgery, premature infant exposure to glucocorticoids, maternal fever during labour, prolonged rupture of membranes, stressful delivery or fetal distress, prolonged labour, perinatal asphyxia or shock, surfactant administration, intraventricular haemorrhage (IVH), and pneumothorax are just a few of the conditions that cause an increase in CRP. Although a high CRP level indicates the presence of infant sepsis, it must be used in conjunction with other markers to make clinical decisions [4]. Serial CRP monitoring 24 to 48 hours after symptoms has been shown to enhance sensitivity in identifying neonatal sepsis, and serial CRP is also used to assess treatment response in sick neonates on antibiotics for the same. CRP's normal serial value is a reliable indicator of the absence of infant sepsis and can be used to identify when medicines should be stopped [13]. 

Highly sensitive C-reactive protein (hs-CRP)

The frequently accepted limit for a significant level of CRP is greater than 6mg/l. Highly sensitive CRP (hs-CRP) is more sensitive than conventional CRP for diagnosing infant sepsis. The cutoff threshold for the hs-CRP assay is lower than that of traditional CRP assays, with a hs-CRP one mg/l value demonstrating higher sensitivity for neonatal infection. According to a recent study from India, when compared to conventional CRP, hs-CRP showed a higher sensitivity [4].

Procalcitonin (PCT)

PCT is a calcitonin prohormone. It is a member of the acute-phase reactant family and is responsible for the vascular response and immunomodulation seen in systemic inflammatory response syndrome (SIRS) [4]. Macrophages and hepatocytes both generate PCT [13]. Furthermore, there appears to be a link between monocyte activation and adherence and PCT production induction [14]. Thyroid para-follicular cells, as well as neuroendocrine cells in the lungs and gut, are other sources of procalcitonin [3]. Within two to four hours of bacterial endotoxin exposure, PCT levels rise rapidly, peaking at six to eight hours and remaining elevated for the next 24 hours [12]. The half-life of PCT is 24-30 hours. When compared to CRP, PCT's rapid rise with the onset of bacterial sepsis makes it a good diagnostic for infant sepsis early diagnosis [13]. Another advantage is that the gestational age of the baby has no effect on the increase in PCT level in bacterial sepsis. Serum PCT levels rise considerably with systemic bacterial infection (EOS and LOS) and necrotizing enterocolitis [4].

PCT has the advantage of maintaining high serum concentrations when compared to other sepsis markers such as tumour necrosis factor-alpha (TNF) and interleukin (IL)-6, making it more useful in the identification of sepsis [4]. However, in non-sepsis situations such as significant burns, severe trauma, acute multiorgan failure, severe medullary thyroid cancer, and major surgeries, procalcitonin levels are also high. As a result, in instances other than sepsis, data must be weighed against other lab values and clinical symptoms [15]. 

Intracranial haemorrhage, neonatal hypoxemia, infants born to mothers with chorioamnionitis in the absence of neonatal infection, prolonged rupture of membranes >18 hours, surfactant administration, very low birth weight (VLBW), premature newborns, birth asphyxia, neonate requiring resuscitation, maternal GBS colonization, and, prenatal antibiotic misuse, postnatal antibiotic use are all examples of the false increase in PCT. PCT cannot correctly identify septic neonates on its own because it has so many reasons to increase incorrectly. As a result, relying on this biomarker risks delaying antibiotic treatment in septic newborns who would otherwise benefit from such potentially life-saving treatment [4]. Using a lower serum PCT cutoff value for the diagnosis of infant sepsis could conceivably increase the test's sensitivity and negative predictive value [16]. 

Finally, due to fungal, gram-positive, gram-negative infections, PCT may act differently in neonatal sepsis. As a consequence, PCT's ability to predict neonatal sepsis is directly or indirectly influenced not only by the clinical manifestations of the neonates enrolled but also by the local microbiological profile in a specified ICU [16].

Serum Amyloid A (SAA)

Proinflammatory cytokines, i.e. interleukin-6 (IL-6) and tumour necrosis factor-alpha, regulate the production of serum amyloid A (SAA). The synthesis of SAA is regulated by proinflammatory cytokines such as interleukin-6 (IL-6) and tumour necrosis factor-alpha [3]. SAA, an early acute phase reactant apolipoprotein, is produced in the liver. SAA can also be found in endothelial cells, smooth muscle cells, and monocytes. SAA production is regulated by IL-1, IL-6, and TNF, and SAA is released in response to infection and injury. Because the level of SAA fluctuates with age, the results should be interpreted after taking the patient's age into consideration. The lowest quantities are seen in umbilical cord blood, whereas the greatest is found in elderly people. Furthermore, SAA contributes to inflammation by causing neutrophils to release IL-8 [4]. 

SAA was the first predictor, whereas CRP and WBC levels can also be employed as prognostic markers in preterm infants' LOS. SAA is a good marker for newborn sepsis detection, and with quick and reliable SAA detection kits, SAA can be a valuable marker for neonatal sepsis detection [4].

Lipopolysaccharide-Binding Protein (LPB)

LPB (lipopolysaccharide-binding protein) is a soluble pattern-recognition molecule that interacts with endotoxin produced by Gram-negative bacteria during infection. LPB is produced by hepatocytes, epithelial cells, and muscle cells, and levels rise rapidly within six to eight hours after the start of acute infection [3]. As a result, LPB is an excellent sensitivity and NPV marker for EOS diagnosis [3]. Another advantage of LPB is that it maintains a consistent level with fewer physiological fluctuations in the first two days after birth and is less affected by obstetrical events [4]. LPB recognizes microbial-associated molecular patterns of bacteria and delivers endotoxin to CD14 immune effector cells in response to infection [4]. LPB binds to the lipopolysaccharide component of gram-negative bacteria, producing a molecule that initiates the early infection response in leucocytes [4]. In comparison to CRP and PCT, the level of LBP peaks early, within six to eight hours after an acute infection (LPS-induced), leading to better sensitivity and NPV for diagnosing EOS [3]. 

Interleukin-6

IL-6 is produced by B and T lymphocytes during the acute phase of an infection, as well as monocytes, endothelial cells, and fibroblasts. IL-6 causes hepatic cells to produce acute phase reactants such CRP [17,18]. With two N-glycosylation sites and four cysteine residues, IL-6 has a 184-amino-acid structure [19]. The advantage of using IL-6 as a sepsis marker is that its concentration rises quickly once bacteremia begins, even before CRP levels rise. The disadvantage of IL-6 is that it has a short half-life and that its level returns to normal within 24 hours of starting antimicrobials, leaving it with a very narrow window of opportunity [20,21]. IL-6 levels in the umbilical cord are greater in newborns with EOS [22,23]. When IL-6 is utilised as an early phase biomarker, studies have demonstrated that it has higher sensitivity and NPV than CRP. When IL-6 is combined with additional sepsis markers as TNF-a and CRP, the sensitivity and NPV for diagnosing EOS improves [4].

Interleukin-8

IL-8, a pro-inflammatory cytokine, is produced by monocytes, macrophages, fibroblasts, and endothelial cells. IL-8 is implicated in the migration and activation of neutrophils during infection [24,25]. It serves as an infection indicator and has a significant link to the severity of the illness. When an infection begins, IL-8 levels rise rapidly, peaking in two to four hours and then rapidly falling in four hours, making it a useful early indicator of infection, analogous to IL-6 [26]. Chemokines have several benefits over CRP as a measure of newborn sepsis, including their quick rise in level (within two to four hours after infection). Nonetheless, important drawbacks include a quick drop in level with treatment commencement (within 24 hours), the necessity for advanced tools for detection, and the unavailability of measuring facilities in the majority of sites, all of which restrict their broad usage [27]. Although the results are promising in terms of understanding the role of IL-8 in the diagnosis of infant sepsis, additional study is needed to improve its use [4].

Tumour Necrosis Factor (TNF-alpha)

It is a proinflammatory chemokine/cytokine produced by activated phagocytes during systemic infection and inflammation. TNF-alpha shares the same characteristics and pharmacokinetics as IL-6 [28], causing a rapid rise 2-4 hours after infection onset. The proinflammatory response to an increase in TNF level is unaffected by the baby's gestational or postnatal age [29]. TNF-alpha concentrations are much higher in septic infants [30,31].

CD11beta

CD11b (Mac-1, CR3), the alpha component of the b2-integrin adhesion molecule, is expressed in small levels in inactivated neutrophils. During an inflammatory reaction, this is engaged in adhesion, diapedesis, and phagocytosis, among other neutrophil functions. A five-minute jump in level indicates a bacterial infection, making it a good diagnostic for recognising infant sepsis early [29]. Weirich et al. looked at neutrophil CD11b expression as a possible E. coli diagnostic marker. They discovered that it was present in all babies with confirmed infection, but not in those who did not have sepsis [32,33]. In clinical studies, CD11b has shown good diagnostic accuracy as a biomarker for infant sepsis; nevertheless, lack of detection capabilities and cost-effectiveness may be important hurdles [4].

sCD 163

CD163 is a soluble glycoprotein receptor on the surface of macrophages that belongs to the SRCR domain family of scavenger receptors. By eliminating the circulating free haemoglobin, haptoglobin helps to decrease the oxidative damage caused by hemolysis. sCD163 binds to gram-positive and gram-negative bacteria, causing proinflammatory cytokines such TNFa, IL-1b, IL-6, and IL-10 to be produced [34]. sCD163 was found to be the most efficient predictor of non-infected versus infected newborns before antibiotics were given [4].

CD 64

The CD64/Fc gamma-receptor is a high-affinity Fc receptor for immunoglobulin G1 and G3 [35], which are expressed in tiny quantities on the surface of inactivated neutrophils [35]. Its level in active neutrophils increases tenfold within four to six hours after the start of bacterial infection. Within a few days after the immune system has eliminated the viral stimulus, expression levels revert to normal [32,36]. CD64 stimulates neutrophilic phagocytosis, an important component of innate immunity [33,34]. Neutrophil CD64 can be utilized in conjunction with other hematologic criteria to assist in the diagnosis of infant sepsis. It also has a number of properties that make it a good biomarker for neonatal sepsis [33,37].

Interleukin-10

Anti-inflammatory cytokines such as IL-10 and TGF- are important inflammatory mediators in sepsis because they help prevent an overly proinflammatory response. Immune system cells such monocytes, macrophages, T and B lymphocytes, and natural killer cells produce IL-1 (NK cells). This cytokine suppresses the manufacturing of proinflammatory mediators including TNF, IL-1, IL-6, IFN, and granulocyte-macrophage colony-stimulating factor (GM-CSF) in immune system cells. Septic shock has been connected to IL-10 in both adults and children. In adults with sepsis, high levels of IL-10 have been associated with a poor prognosis and have been shown to be a strong predictor of septic shock severity and fatality. Sepsis in newborns has a direct influence on treatment and prognosis. An optimum IL-10 response, on the other hand, has been shown to protect against SIRS, and a high IL-6/IL-10 ratio has been seen in patients with a poor prognosis. Severe late-onset infant sepsis has been associated with a high IL-10/TNF ratio. An imbalance of pro-inflammatory and anti-inflammatory cytokines appears to be linked to the severity and prognosis of infant sepsis. As a result, using cytokines as biomarkers for infant sepsis is both rational and necessary, because early diagnosis of neonatal sepsis has a direct influence on treatment and prognosis [38].

Pentraxin 3 

Pentraxin 3 (PTX3), like CRP, is a soluble pattern recognition receptor expressed by monocytic cells (monocyte/macrophage and dendritic cells), endothelial cells, and neutrophils [39]. Its primary activities include microbe identification, complement activation, and opsonization. PTX3 is stored in neutrophils, primarily in specific granules, and to a lesser extent in azurophilic and gelatinase granules. PTX3 is released into the extracellular space and localised in neutrophil extracellular traps (NETS) in response to stimuli such as pathogens and inflammatory cytokines (e.g., TNF-, IL-1) [40].

A study found that deceased neonates with sepsis had considerably higher serum levels of PTX3 than survivors, suggesting that PTX3 could be utilised as a predictive biomarker for neonatal sepsis. In addition, researchers observed that PTX3 concentrations were considerably higher in severely ill children than controls in a recent study [40]. Platelet count was also found to be substantially lower in newborns with sepsis, and PTX3 was found to be adversely linked with platelet count in studies. This was in agreement with Mauri et al., who demonstrated that PTX3 was substantially associated with blood platelet count in 90 patients with sepsis in their investigation [41]. Classic pentraxins, such as CRP and serum amyloid P component (SAP), are frequently measured and recognized as indicators of inflammation; however, they provide little information regarding risk assessment and patient prognosis in sepsis caused by several infections. However, patient data suggest that raised PTX3 levels are linked to higher illness severity and fatality rates [39]. PTX3 has also been found in a few studies to be a substantially accurate and more specific predictor of patient outcome and disease severity than more common indicators such as TNF, CRP, and IL-6 [1]. As proven in vitro, PTX3 may lead to coagulopathy through tissue factor (TF) upregulation on the surface of monocytes and vascular endothelial cells. In addition to being a more reliable measure of illness severity in cases of sepsis, PTX3 levels, in combination with other coagulation function assays, such as platelet count, PT, and aPTT, may also be beneficial in identifying patients in need of anticoagulation medications [39].

However, few studies showed that pentraxin 3, compared to procalcitonin and CRP, did not have a diagnostic advantage in predicting sepsis in clinical practice. In conclusion, pentraxin 3 seems like a promising prognostic marker in neonatal sepsis [42]. PTX3 could be used as a new biomarker to predict the severity and prognosis of a number of inflammatory and viral diseases [39]. Additional research using more extensive patient series is needed to support this marker’s sensitivity, specificity, and utility for disease progression [42]. 

Angiopoietins

Angiopoietins are angiogenic factors that belong to a glycoproteins family (growth factors) that act predominantly on the vasculature to regulate blood vessel development and stability. Angiopoietin-1 (Ang-1), angiopoietin-2 (Ang-2), angiopoietin-3 (Ang-3), and angiopoietin-4 (Ang-4) are the four angiopoietins that have been identified. Ang-1 is mainly produced in the pericytes and smooth muscle cells surrounding the endothelial cell monolayer. Endothelial cells produce Ang-2, kept in Weibel-Palade bodies for fast release when exposed to inflammatory stimuli. Ang-3 is found in mouse tissues, while Ang-4 is only found in human lungs at significant concentrations. Ang-1 and Ang-2 are antagonistic ligands of the Tie-2 receptor - a vascular tyrosine kinase receptor expressed mainly in the endothelial cells [43].

In typical situations, the serum levels of Ang-1 surpass those of Ang-2. This allows Ang-1 to bind to Tie-2 receptors and therefore trigger pro-survival pathways while inhibiting pro-inflammatory pathways. Conversely, inflammation leads to exocytosis of Weibel-Palade bodies and Ang-2 release, allowing it to selectively bind the Tie-2 receptor, enhance pro-inflammatory and pro-thrombotic pathways, and microvascular leak. Ang-1 and -2 appear to be promising as a predictive sepsis biomarker, representing the endothelium's direct status linked to disease severity and prognosis. Ang-1 levels at admission to the NICU may predict the outcome, and Ang-2 levels, which show a similar pattern to TNF-alpha and IL-6, may be interesting for assessing septic infants [43].

SuPar

Several studies showed a link between SuPAR and mortality in individuals with malaria, TB, and HIV infection [44]. SuPAR, or soluble urokinase-type plasminogen activator receptor, has been postulated as a promising biomarker of immunological activation. Many cell types, including monocytes and macrophages, produce a urokinase-type plasminogen activator, responsible for migrating inflammatory cells from the bloodstream into tissues [45].

SuPAR levels rise in critically ill individuals, although this rise is not particular to sepsis. As a result, suPAR is ineffective as a diagnostic biomarker. On the other hand, it has been shown to have prognostic value in many recent studies and is a promising biomarker in this group [45]. The median serum value of the soluble urokinase-type plasminogen activator receptor in an investigation of 314 HIV-1 infected participants was 3.69 ng/ml. Individuals with greater viral loads, lower CD4 counts, and a greater incidence of AIDS-related death had elevated serum levels. CD4 count and SuPAR levels had a weak but substantial negative association, whereas viral load and suPAR levels had a weak positive correlation [44].

In conclusion, high levels of suPAR have been linked to outcome and morbidity in NICU patients, implying that it could be used as a prognostic biomarker; additionally, concentrations greater than ten ng/ml have been linked to mortality in many investigations [44].

*Soluble Triggering Receptor Expressed on Myeloid Cell-1*​ (​​​​​​*TREM-1)*

Triggering receptor expressed on myeloid cell-1 (TREM1) is a transmembrane glycoprotein identified on neutrophil and monocyte surfaces. In fungal and bacterial infections, it increases the production of several pro-inflammatory cytokines via the activation of the DNA activating protein 12 (DAP12) signaling pathway. Soluble TREM-1 (sTREM-1) is a 17-kDa fragment synthesized by metalloproteinase cleavage that can be found in cerebrospinal fluid, bronchoalveolar lavage, and blood [46].

Earlier, scientists conducted a study to see if sTREM-1 could be used as a diagnostic and prognostic marker in neonatal sepsis. They found that baseline sTREM-1 levels were significantly higher in culture-proven and culture-negative sepsis compared to controls, with no significant difference between the septic groups. In addition, some researchers discovered that sTREM-1 levels in septic newborns were considerably more critical than in non-septic neonates [4]. sTREM-1 is a promising inflammatory biomarker with diagnostic and prognostic potential, according to emerging data [46]. However, there is inadequate evidence to support the use of sTREM in the diagnosis and follow-up of pediatric sepsis, according to a recent systematic review involving four newborn trials [4].

Inter Alpha Inhibitors

Inter alpha inhibitor proteins belong to serine protease inhibitors involved in various biological processes, including tumour invasion, extracellular matrix integrity, inflammation, and wound healing [4].

Researchers explored inter alpha inhibitors as a diagnostic marker in a group of preterm and term newborns. High circulation amounts of inter alpha inhibitor were identified in newborn infants compared to those observed in adults, and the levels were unaffected by gestational age. Moreover, uninfected infants had considerably greater inter alpha inhibitor concentrations than affected infants [47].

In addition, after four to 12 days of antibiotic medication, longitudinal studies of ill neonates revealed that inter alpha inhibitor levels had returned to normal and hence can be used as a prognostic marker to keep track of treatment's effectiveness [4,47]. Few authors concluded that inter alpha inhibitor is a more reliable diagnostic biomarker for sepsis than other tests. However, more research is needed to determine the relevance of inter alpha inhibitor as a biomarker for neonatal sepsis [47].

Mannose-Binding Lectin

Mannose-binding lectin (MBL) is a component of innate immunity; it is especially crucial in neonates who lack fully established adaptive immunity. MBL circulates in the bloodstream and binds to carbohydrate structures on the surface of various bacteria, providing first-line immunity. Some writers have observed a link between low MBL levels and infection susceptibility in healthy children and adults, although the evidence remains inconclusive [48]. According to recent investigations, infected newborns had lower serum MBL levels than control infants. This confirms previous studies that infected newborns have lower MBL levels [48].

In conclusion, low MBL serum levels could be used as a sensitive and specific marker in newborn infants to predict sepsis, septic shock, and associated clinical consequences. MBL levels measured at admission may aid in identifying newborns at high risk of sepsis in the NICU [48].

Presepsin

Presepsin is a soluble CD14 fragment that has been shown to predict adult sepsis. Given its high sensitivity and specificity, the findings of a metaanalysis imply that presepsin could be a potential biomarker in newborn sepsis. Presepsin's significance as a sepsis marker is further underlined by the fact that, unlike standard laboratory tests, the values of its serum are unaffected by a variety of prenatal factors associated with noninfectious illnesses [49].

Furthermore, three investigations have demonstrated that serum presepsin declines over time following antibiotic treatment, implying that it may play a role in evaluating therapeutic response during the recovery period. Finally, presepsin levels should be evaluated alongside clinical factors and other laboratory tests to create a combined model that gives quality diagnostic results [49].

Prospects, developments, and limitations of novel biomarkers in the diagnosis and treatment of neonatal sepsis

The detection of numerous potential biomarkers could lead to improved evaluation and treatment of newborn sepsis in the future. Even with the existence of several promising candidate biomarkers, no distinct biomarker, a group of biomarkers, or scoring system can be used exclusively at this time [50]. Emerging biomarkers will need to be individually and prospectively evaluated, with large cohorts in multidisciplinary trials ideally enrolled [51]. Future research on biomarkers should emphasize identifying, creating, and refining quick, sensitive, and specific diagnostic techniques that can accurately screen for and detect all pathogens that cause newborn sepsis [51]. The limitations of this research are the paucity of suitable "disease-exposed" samples and controls and skilled analysis of the complex datasets. In their current form, the studies are time intensive and ineffective for making early decisions about sepsis therapy [50].

Novel approaches such as proteomic and metabolomic studies look at how proteins or metabolites function in a sample and can be used to diagnose neonatal bacterial and fungal sepsis [4]. The proteomic analysis detected two promising biomarkers - pro apolipoprotein CII and a desarginine variant of serum amyloid A (SAA), which effectively guided antimicrobial management and excluded sepsis with a 100% negative predictive value [51]. In comparison to other biomarkers like CRP and IL-6, which have inadequate sensitivities at different stages of an illness, SAA was found to be a reliable screening sign for the first 24 hours following infection start, thus raising its potential relevance as a biomarker. In addition, alpha-1 antitrypsin, fibronectin, haptoglobin, lactoferrin, and neopterin are some biomarkers that could be useful with other sepsis indicators [50]. This technology was effectively used in the interpretation of amniotic fluid of pregnant women with the risk of preterm delivery and to isolate microorganisms before the signs and symptoms of neonatal sepsis appear [28]. Recent breakthroughs in proteomics have opened up new possibilities for finding biomarkers and creating protein profiles that can help predict amniotic fluid inflammation and early neonatal sepsis in as little as three hours [52].

Metabolomics and nuclear magnetic resonance imaging may be helpful to measure concentrations of metabolites such as acylcarnitines and glycerophosphatidylcholines, which appeared to be substantially increased in sepsis compared to SIRS, which can be used to distinguish infectious from non-infectious systemic inflammation. But, the inability to accurately determine whether the proteins found correlate with sepsis, as well as the difficulties of detecting low amounts in the blood, limit this technique [51].

Various approaches have been utilised to measure the number of biomarkers for neonatal sepsis, including nephelometric and turbidimetric technologies, ELISA, and blood cultures. Unfortunately, these approaches have drawbacks such as low sensitivity, high cost, and long processing times. Therefore, new guidelines for identifying biomarkers have been developed in recent years, such as quartz crystal microbalance, surface plasmon resonance (SPR), piezoelectric microcantilevers, electrochemistry, and microfluidics. Out of these methods, electrochemical assays are the most accessible and accurate, with higher sensitivity, shorter response times, point-of-care convenience, low cost, and user flexibility [53].

Nanotechnology-based electrochemical sensing combined with integrated electronics can also detect biomarkers. They are used to make devices for sensing biological, chemical, or mechanical forces induced by changes in the target or analyte parameters at the nanoscale level. They help improve specificity while detecting the biomarker of interest. Biomarkers with low concentrations can be identified from samples due to the larger surface area to volume ratio of nanoparticles [53].

To advance to point-of-care testing in the clinical world, additional steps are needed, such as standardization of technologies and platforms, demonstration of clinical benefit in the management process, or on patient outcome, workflow and efficiency improvements, and proved cost-effectiveness and must be aligned with broader societal goals of improving data on the etiologies of newborn sepsis, especially in low- and middle-income countries [51]. In the future, efforts will be made to establish a multiplexed platform for different biomarkers with a multiparameter screening of newborn sepsis [53].

## Conclusions

Traditional haematological and microbiological techniques used to detect newborn sepsis remain ineffective in the face of high mortality and severe morbidity. The possible utilization of novel technological advances and changing awareness of present biomarkers’ strengths and limits has improved biomarkers that offer early specific and reliable identification of the neonate at the risk of the infection. In addition, a better understanding of the neonatal immune systems’ capabilities in response to infection has led to the discovery of several intriguing prospective biomarkers that could lead to improved neonatal sepsis diagnosis, treatment, and prognosis in the future. Although no single biomarker serves as an ideal marker, the most interesting and reliable biomarkers are soluble CD14 subtype presepsin (SCD14-ST), lipopolysaccharide-binding protein, angiopoietin (Ang)-1 and -2, soluble form of urokinase-type plasminogen activator receptor (SuPAR), platelet-activating factor and calprotectin. Metabolomics marks the transition from a descriptive to a predictive science, turning research into actual clinical use. 

## References

[1] Hincu MA, Zonda GI, Stanciu GD, Nemescu D, Paduraru L (2020). Relevance of Biomarkers Currently in Use or Research for Practical Diagnosis Approach of Neonatal Early-Onset Sepsis. Children (Basel).

[2] Memar MY, Alizadeh N, Varshochi M, Kafil HS (2019). Immunologic biomarkers for diagnostic of early-onset neonatal sepsis. J Matern Fetal Neonatal Med.

[3] Chauhan N, Tiwari S, Jain U (2017). Potential biomarkers for effective screening of neonatal sepsis infections: An overview. Microb Pathog.

[4] Sharma D, Farahbakhsh N, Shastri S, Sharma P (2018). Biomarkers for diagnosis of neonatal sepsis: a literature review. J Matern Fetal Neonatal Med.

[5] Rashwan NI, Hassan MH, Mohey El-Deen ZM, Ahmed AE (2019). Validity of biomarkers in screening for neonatal sepsis - A single center -hospital based study. Pediatr Neonatol.

[6] Bedford Russell AR, Kumar R (2015). Early onset neonatal sepsis: diagnostic dilemmas and practical management. Arch Dis Child Fetal Neonatal Ed.

[7] Hengst JM (2003). The role of C-reactive protein in the evaluation and management of infants with suspected sepsis. Adv Neonatal Care.

[8] Chiesa C, Panero A, Osborn JF, Simonetti AF, Pacifico L (2004). Diagnosis of neonatal sepsis: a clinical and laboratory challenge. Clin Chem.

[9] Chiesa C, Pellegrini G, Panero A, Osborn JF, Signore F, Assumma M, Pacifico L (2003). C-reactive protein, interleukin-6, and procalcitonin in the immediate postnatal period: influence of illness severity, risk status, antenatal and perinatal complications, and infection. Clin Chem.

[10] Døllner H, Vatten L, Austgulen R (2001). Early diagnostic markers for neonatal sepsis: comparing C-reactive protein, interleukin-6, soluble tumour necrosis factor receptors and soluble adhesion molecules. J Clin Epidemiol.

[11] Hedegaard SS, Wisborg K, Hvas AM (2015). Diagnostic utility of biomarkers for neonatal sepsis--a systematic review. Infect Dis (Lond).

[12] Cantey JB, Lee JH (2021). Biomarkers for the diagnosis of neonatal sepsis. Clin Perinatol.

[13] Shah BA, Padbury JF (2014). Neonatal sepsis: an old problem with new insights. Virulence.

[14] Delanghe JR, Speeckaert MM (2015). Translational research and biomarkers in neonatal sepsis. Clin Chim Acta.

[15] Bell SG (2017). Procalcitonin and neonatal sepsis: is this the biomarker we are looking for?. Neonatal Netw.

[16] Vouloumanou EK, Plessa E, Karageorgopoulos DE, Mantadakis E, Falagas ME (2011). Serum procalcitonin as a diagnostic marker for neonatal sepsis: a systematic review and meta-analysis. Intensive Care Med.

[17] Kishimoto T (1989). The biology of interleukin-6. Blood.

[18] Hodge G, Hodge S, Han P, Haslam R (2004). Multiple leucocyte activation markers to detect neonatal infection. Clin Exp Immunol.

[19] Hirano T, Yasukawa K, Harada H (1986). Complementary DNA for a novel human interleukin (BSF-2) that induces B lymphocytes to produce immunoglobulin. Nature.

[20] Raynor LL, Saucerman JJ, Akinola MO, Lake DE, Moorman JR, Fairchild KD (2012). Cytokine screening identifies NICU patients with Gram-negative bacteremia. Pediatr Res.

[21] Küster H, Weiss M, Willeitner AE (1998). Interleukin-1 receptor antagonist and interleukin-6 for early diagnosis of neonatal sepsis 2 days before clinical manifestation. Lancet.

[22] Smulian JC, Vintzileos AM, Lai YL, Santiago J, Shen-Schwarz S, Campbell WA (1999). Maternal chorioamnionitis and umbilical vein interleukin-6 levels for identifying early neonatal sepsis. J Matern Fetal Med.

[23] Smulian JC, Bhandari V, Campbell WA, Rodis JF, Vintzileos AM (1997). Value of umbilical artery and vein levels of interleukin-6 and soluble intracellular adhesion molecule-1 as predictors of neonatal hematologic indices and suspected early sepsis. J Matern Fetal Med.

[24] Reinsberg J, Dembinski J, Dorn C, Behrendt D, Bartmann P, van Der Ven H (2000). Determination of total interleukin-8 in whole blood after cell lysis. Clin Chem.

[25] Franz AR, Sieber S, Pohlandt F, Kron M, Steinbach G (2004). Whole blood interleukin 8 and plasma interleukin 8 levels in newborn infants with suspected bacterial infection. Acta Paediatr.

[26] Resch B, Gusenleitner W, Müller WD (2003). Procalcitonin and interleukin-6 in the diagnosis of early-onset sepsis of the neonate. Acta Paediatr.

[27] Mehr S, Doyle LW (2000). Cytokines as markers of bacterial sepsis in newborn infants: a review. Pediatr Infect Dis J.

[28] Mishra UK, Jacobs SE, Doyle LW, Garland SM (2006). Newer approaches to the diagnosis of early onset neonatal sepsis. Arch Dis Child Fetal Neonatal Ed.

[29] Ng PC, Lam HS (2006). Diagnostic markers for neonatal sepsis. Curr Opin Pediatr.

[30] Berner R, Niemeyer CM, Leititis JU (1998). Plasma levels and gene expression of granulocyte colony-stimulating factor, tumor necrosis factor-alpha, interleukin (IL)-1beta, IL-6, IL-8, and soluble intercellular adhesion molecule-1 in neonatal early onset sepsis. Pediatr Res.

[31] Ng PC, Cheng SH, Chui KM (1997). Diagnosis of late onset neonatal sepsis with cytokines, adhesion molecule, and C-reactive protein in preterm very low birthweight infants. Arch Dis Child Fetal Neonatal Ed.

[32] Weirich E, Rabin RL, Maldonado Y, Benitz W, Modler S, Herzenberg LA, Herzenberg LA (1998). Neutrophil CD11b expression as a diagnostic marker for early-onset neonatal infection. J Pediatr.

[33] Adib M, Ostadi V, Navaei F, Saheb Fosoul F, Oreizi F, Shokouhi R, Bakhshiani Z (2007). Evaluation of CD11b expression on peripheral blood neutrophils for early detection of neonatal sepsis. Iran J Allergy Asthma Immunol.

[34] Graversen JH, Madsen M, Moestrup SK (2002). CD163: a signal receptor scavenging haptoglobin-hemoglobin complexes from plasma. Int J Biochem Cell Biol.

[35] Lv B, Huang J, Yuan H, Yan W, Hu G, Wang J (2014). Tumor necrosis factor-α as a diagnostic marker for neonatal sepsis: a meta-analysis. ScientificWorldJournal.

[36] Nupponen I, Andersson S, Järvenpää AL, Kautiainen H, Repo H (2001). Neutrophil CD11b expression and circulating interleukin-8 as diagnostic markers for early-onset neonatal sepsis. Pediatrics.

[37] Sprong T, Peri G, Neeleman C, Mantovani A, Signorini S, van der Meer JW, van Deuren M (2009). Pentraxin 3 and C-reactive protein in severe meningococcal disease. Shock.

[38] Machado JR, Soave DF, da Silva MV (2014). Neonatal sepsis and inflammatory mediators. Mediators Inflamm.

[39] Ketter P, Yu JJ, Cap AP, Forsthuber T, Arulanandam B (2016). Pentraxin 3: an immune modulator of infection and useful marker for disease severity assessment in sepsis. Expert Rev Clin Immunol.

[40] Fahmey SS, Mostafa N (2019). Pentraxin 3 as a novel diagnostic marker in neonatal sepsis. J Neonatal Perinatal Med.

[41] Mauri T, Bellani G, Patroniti N (2010). Persisting high levels of plasma pentraxin 3 over the first days after severe sepsis and septic shock onset are associated with mortality. Intensive Care Med.

[42] Battal F, Bulut Ö, Yıldırım Ş, Aylanç H, Kaymaz N, Uysal S (2019). Serum pentraxin 3 concentration in neonatal sepsis. J Pediatr Infect Dis.

[43] Mussap M, Cibecchini F, Noto A, Fanos V (2013). In search of biomarkers for diagnosing and managing neonatal sepsis: the role of angiopoietins. J Matern Fetal Neonatal Med.

[44] Donadello K, Scolletta S, Covajes C, Vincent JL (2012). suPAR as a prognostic biomarker in sepsis. BMC Med.

[45] Sandquist M, Wong HR (2014). Biomarkers of sepsis and their potential value in diagnosis, prognosis and treatment. Expert Rev Clin Immunol.

[46] Bellos I, Fitrou G, Daskalakis G, Thomakos N, Papantoniou N, Pergialiotis V (2018). Soluble TREM-1 as a predictive factor of neonatal sepsis: a meta-analysis. Inflamm Res.

[47] Lam HS, Ng PC (2008). Biochemical markers of neonatal sepsis. Pathology.

[48] Wahab Mohamed WA, Saeed MA (2012). Mannose-binding lectin serum levels in neonatal sepsis and septic shock. J Matern Fetal Neonatal Med.

[49] Bellos I, Fitrou G, Pergialiotis V, Thomakos N, Perrea DN, Daskalakis G (2018). The diagnostic accuracy of presepsin in neonatal sepsis: a meta-analysis. Eur J Pediatr.

[50] Mally P, Xu J, Hendricks-Muñoz K (2014). Biomarkers for neonatal sepsis: Recent developments. Res Rep Neonatol.

[51] Iroh Tam PY, Bendel CM (2017). Diagnostics for neonatal sepsis: current approaches and future directions. Pediatr Res.

[52] Arnon S, Litmanovitz I (2008). Diagnostic tests in neonatal sepsis. Curr Opin Infect Dis.

[53] Balayan S, Chauhan N, Chandra R, Kuchhal NK, Jain U (2020). Recent advances in developing biosensing based platforms for neonatal sepsis. Biosens Bioelectron.

